# Supplementary material for: A Possible Natural and Inexpensive Substitute for Lapis Lazuli in the Frederick II Era: The Finding of Haüyne in Blue Lead-Tin Glazed Pottery from Melfi Castle (Italy)
Source: Molecules. 2023 Feb 6;28(4):1546. doi: 10.3390/molecules28041546 (PMC9965324; doi:10.3390/molecules28041546)
Supplement: Supplementary file 1 [file molecules-28-01546-s001.zip › SM1 Supplementary materials 1_proof final AR2.pdf]

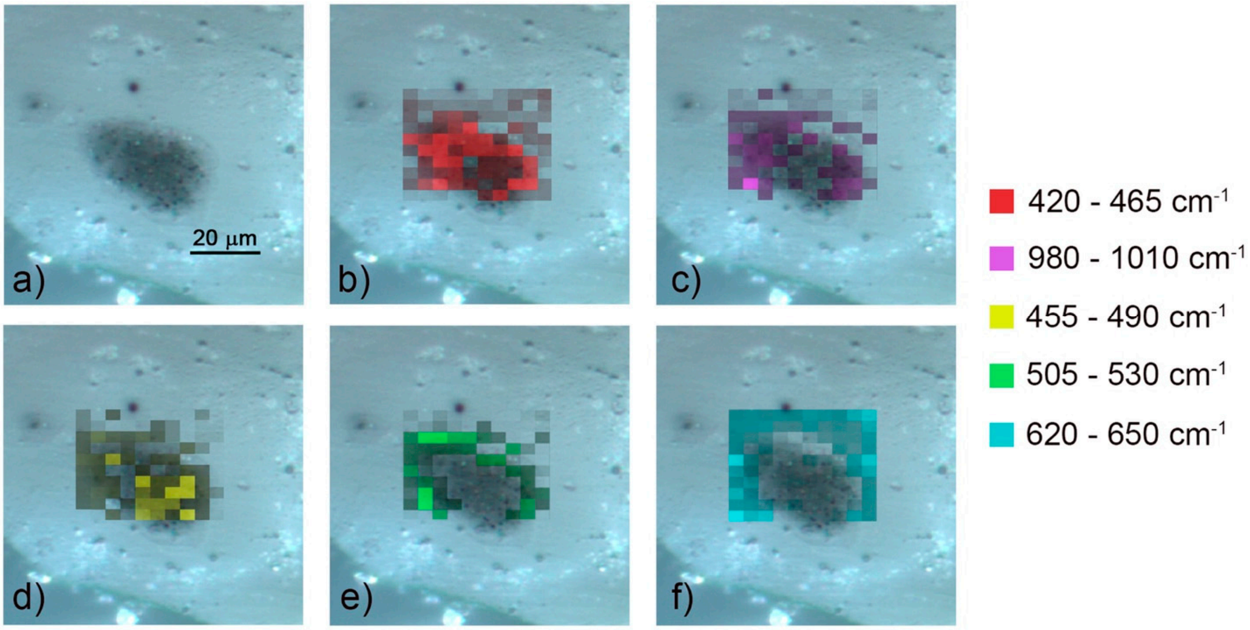

Figure S1: Optical image of haüyne 3 (a) and results of the respective Raman mapping according to 420-465 cm^{-1} (b), 980-1010 cm^{-1} (c), 455-490 cm^{-1} (d), 505-530 cm^{-1} (e) and 620-650 cm^{-1} (f) spectral regions.

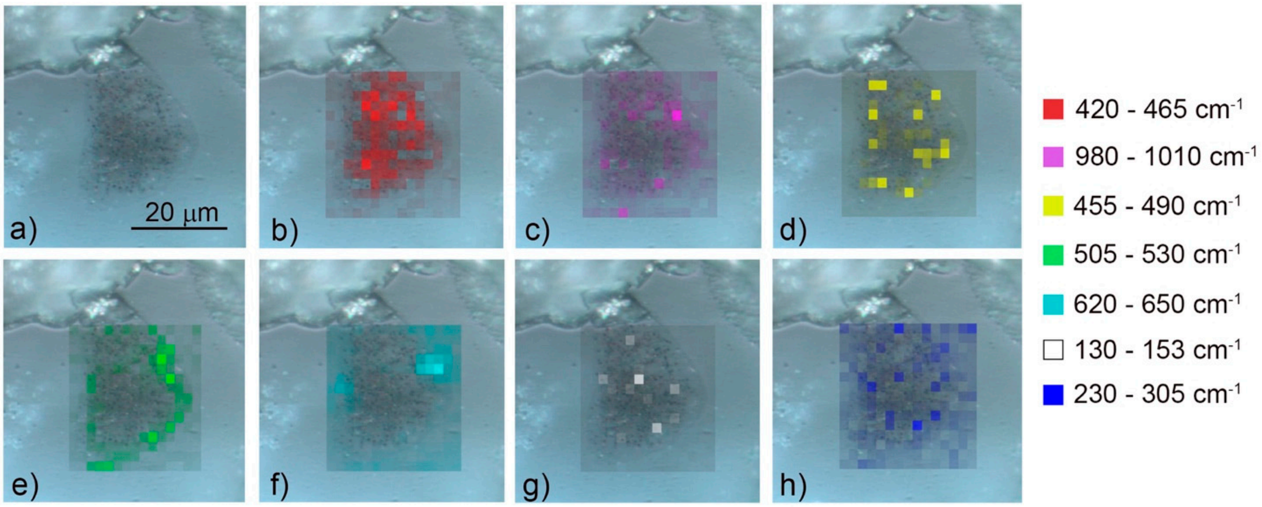

Figure S2: Optical image of haüyne 5 (a) and results of the respective Raman mapping according to 420-465 cm^{-1} (b), 980-1010 cm^{-1} (c), 455-490 cm^{-1} (d), 505-530 cm^{-1} (e), 620-650 cm^{-1} (f), 130-153 cm^{-1} (g) and 230-305 cm^{-1} (h) spectral regions.
