# Supplementary material for: A Possible Natural and Inexpensive Substitute for Lapis Lazuli in the Frederick II Era: The Finding of Haüyne in Blue Lead-Tin Glazed Pottery from Melfi Castle (Italy)
Source: Molecules. 2023 Feb 6;28(4):1546. doi: 10.3390/molecules28041546 (PMC9965324; doi:10.3390/molecules28041546)
Supplement: Supplementary file 1 [file molecules-28-01546-s001.zip › SM2 Supplementary materials 2_proof final AR2.pdf]

Historical sources of lapis lazuli

Historical sources were located in very inaccessible places, such as the Afghan and Pamir mountains, and stones were transported thousands of kilometres. Knowledge of the trade routes used in antiquity is still largely incomplete: this is especially true for ancient contexts where there is an absence or scarcity of written evidence [41]. Although the Badakhshan mines in Afghanistan (most famously Sar-e Sang) are widely regarded as the only sources of lapis lazuli in antiquity [42] other sources have been considered: Tajikistan (Lyadzhuar Dara, Pamir Mountains), Pakistan (Chagai Hills), Siberia (Irkutsk, near Lake Baikal), Iran [43] and Egypt (Sinai) [44]. The last two possibilities are not geologically confirmed and their interpretations are still debated [43,45,46], so the origin of ancient lapis lazuli is still an open question. Other sources [47] are: Mazanderan (but there are many doubts about this and it seems rather unlikely [48]), Dizmar in Azerbaijan and Kerman (there are doubts about the presence of metamorphic rocks and no evidence of lapis lazuli [43,49]). There are also references to other sources [45], particularly northern China and Tibet.

Sodalite-bearing rocks are not widespread; in particular haüyne bearing rocks are mainly present in Canary Islands (Tenerife), Cape Verde (Santo Antao), Ecuador (Sumaco volcano), USA (Colorado, Cripple Creek; Montana, Winnett), Tahiti, Tanzania (Oldoinyo Lengai), Tibet (North Qiangtang), France (Massif Central), Germany (Laacher See, the unique outcrop with gem quality haüyne in the world) and finally, the numerous and widespread outcrops present in Italy (Vulsini, Vico, Alban hills, Ventotene-Santo Stefano, Vulture, Vesuvius) [6,7].

SEM-EDS operative conditions

SEM operative conditions were: 15 kV accelerating potential, 200 pA probe current, about 25,000 output cps as average count rate on the whole spectrum, counting time 50 s for haüyne and more than 100 minutes for enamel, and 8,5 mm working distance.

X-ray intensities were converted into quantitative wt% (w/w) oxides by XPP (eXtended Pouchou and Pichoir) software support, a specific $\Phi(\varrho z)$ correction method developed by Pouchou and Pichoir [50,51] correction scheme, granted as quantitative calculation program

by Oxford-Link Analytical (U.K.).

Analytical precision was 0.5% for concentrations > 15 wt.%, 1% for concentrations of about 5 wt.%, and < 15% for concentrations near the detection limit. For the analyses of the enamel, in which some element (Ti, Cu and Co) concentrations is very near the detection limit, the acquisition time of the detector was set at hundreds of seconds (100 - 500 s). The accuracy of the analytical data was also checked analyzing lazurite of two lapis lazuli samples (Sar-e Sang outcrops of Afghanistan and Condoriaco outcrops of Chile) of the Earth Science Museum of the Bari University and also standard manufactured by Micro-Analysis Consultants Ltd. (U.K.). Crystal-chemical formula of lazurite and haüyne was calculated as suggested by Hogart and Griffin [23].

It is important to point out that microanalyses of sodalite group minerals, when performed with a scanning electron microscope (SEM) or with an Electron Probe Micro Analysis (EPMA), must take into account the possible evaporation of the sample [1,23]. To perform sodalite microanalyses with a WD microspectrometer, as that mounted on Electron Micro Probe Analyser (EPMA), a correct procedure can be realized "using a defocussed beam (approx. 20 μm diameter) and moving the sample under the beam during analysis" [23], to minimize the possible evaporation of the mineral.

Alternatively ED spectrometers allow to analyse small crystals (down to 5 μm in size) or very little part of the same phase (e.g. core and rim), belonging to the sodalite group: this is possible because the ED spectrometer need a lower electron probe current (down to 50 pA) compared to WD spectrometer; the very low probe current necessary for ED microanalyses minimizes vaporization of the sample [1] and at the same time reduce the electron beam diameter [52,53] and consequently also the volume of X-ray investigation (escape volume). During ED analyses presented in this paper, a probe diameter of 200 nm (the probe diameter was calculated according to [53] and using a probe current of 200 pA) was used with an estimated X-ray escape volume of about 5 μm^3 for lazurite and haüyne.

In the case of the chemical characterization of the little blue minerals found in the M22 sample was very important to use a very small electron beam diameter, for SEM images, that generate a very small X-ray investigation volume, to analyze little minerals containing

also very little inclusions of other phases: in this case the use of a SEM coupled with an ED spectrometer was a compulsory choice.
